# Preliminary evidence for altered brain-heart coherence during anxiogenic movies

**DOI:** 10.1162/imag_a_00156

**Published:** 2024-05-02

**Authors:** Peter A. Kirk, Oliver J. Robinson

**Affiliations:** Institute of Cognitive Neuroscience, University College London, London, United Kingdom; Experimental Psychology, University College London, London, United Kingdom; Clinical, Educational and Health Psychology, University College London, London, United Kingdom

**Keywords:** anxiety, cardiac, movie fMRI, naturalistic

## Abstract

During states of anxiety, fundamental threat circuitry in the brain can increase heart rate via alterations in autonomic balance (increased sympathetic activity and parasympathetic withdrawal) and may serve to promote interoceptive integration and awareness of cardiac signals. Moreover, evidence indicates pathological anxiety could be associated with increased communication between the brain and the heart. Yet, this phenomenon remains not well understood. For instance, studies in this area have been conducted within the confines of tightly controlled experimental paradigms. Whether anxiety impacts brain-heart communication outside of such experimental settings, and in relatively more naturalistic contexts, is less clear. Here, we used a suspenseful movie fMRI paradigm to study induced anxiety (n = 29 healthy volunteers; Caltech Conte dataset;Kliemann et al., 2022). We predicted that brain responses across an anxiety-relevant “defensive response network” (amygdala, hypothalamus, periaqueductal gray, bed nucleus of the stria terminalis, dorsomedial prefrontal cortex, ventromedial prefrontal cortex, subgenual anterior cingulate, and anterior insula;Abend et al., 2022) would show increased coherence with heart rate as participants watched a suspenseful movie clip compared to a non-suspenseful movie clip. Counter to our predictions, we found decreased coherence between heart rate and brain responses during increased anxiety, namely in amygdala-prefrontal circuitry. We suggest these alterations may be underpinned by parasympathetic withdrawal and/or decreased interoceptive awareness during suspenseful movie-watching.

## 1 Introduction

One function of anxiety is to promote the detection, identification, and avoidance of harm (Mobbs et al., 2015). When under a state of induced anxiety, neural systems can tune perceptual and cognitive processes to promote such vigilance (attention to threat-relevant stimuli;Eysenck et al., 2007). Underlying this is a core network of cortico-subcortical brain circuitry, which includes the amygdala; bed nucleus of the stria terminalis (BNST); hypothalamus; periaqueductal gray (PAG); anterior insula; dorsomedial prefrontal cortex (dmPFC); subgenual anterior cingulate cortex (sgACC); and anterior ventromedial prefrontal cortex (vmPFC), all of which serve a multitude of functions (Chavanne & Robinson, 2021;Grupe & Nitschke, 2013). Here, we refer to this as the “defensive response network” (Abend et al., 2022). By no means do these regions necessarily constitute the sole circuitry responsible for anxiety (for instance, other regions such as the orbitofrontal cortex have been implicated,Canteras et al., 2010). However, this network captures some of the fundamental systems responsible for driving states of anxiety. A plethora of research in recent decades has focused on how these systems link anxiety to processes such as attention, reward, punishment, and memory (Robinson et al., 2013). At the same time, we know that anxiety also induces—and is influenced by—alterations in peripheral physiology; most notably, the autonomic nervous system.

At the outset of modern psychology, the central nervous system was not always seen as the fundamental basis of anxiety. Instead, peripheral physiology was posited to be the biological driver of affect (Dewey, 1894;James, 1894). Since then, the role of peripheral physiology in anxiety has often been studied as a separate line of inquiry to direct studies of brain activity. Research has established a link between anxiety and activity across branches of the autonomic nervous system (often summarized as increased sympathetic activation and/or parasympathetic withdrawal), using proxies such as heart rate, heart rate variability, and skin conductance (Friedman & Thayer, 1998;Hodges & Spielberger, 1966;Lader, 1967). Whether different proxies, such as heart rate variability, represent different underlying autonomic mechanisms remains unclear (seePham et al., 2021). Nonetheless, dominance of the sympathetic branch of the autonomic system can assist an individual during “fight-flight”-like contexts (whether that be through increased sympathetic activity or parasympathetic withdrawal); for instance, by providing increased blood flow to support musculature for physical exertion (e.g., fleeing;McCorry, 2007). As such, when anxiety is induced (e.g., through threat of shock or horror movies), increases in heart rate are typically observed (de Groot et al., 2020;Smith et al., 1985).

A “central autonomic network” in the brain has been proposed to be responsible for such regulation of autonomic balance (Benarroch, 1993). This includes: insula, dorsomedial and ventromedial prefrontal cortices (including orbitofrontal cortex), amygdala, bed nucleus of the stria terminalis, thalamus, hypothalamus, basal ganglia, periaqueductal gray, and brain stem (Benarroch, 1993;Quadt et al., 2022;Sklerov et al., 2019). Many of these regions overlap with the aforementioned “defensive response network,” but three regions have been consistently linked with emotion-induced regulation of autonomic responses: amygdala, dmPFC, and insula (Critchley, 2005;Thayer et al., 2012;Wager et al., 2009). Moreover, the extent to which these circuits evoke autonomic change in response to induced*state*anxiety may interact with individual differences in features such as age, sex, and*trait*vulnerability to anxiety (Abend et al., 2022;Alvares et al., 2013;Makovac et al., 2016;Mutz et al., 2022). Therefore, anxiety-relevant brain systems (“defensive response network”), which have largely been discussed in terms of perceptual processes, such as threat vigilance, may also be responsible for driving alterations in autonomic balance.

While much research conceptually embeds findings in regard to top-down autonomic entrainment (e.g., brain signaling to the heart), the relationship between central and peripheral nervous systems is reciprocal (e.g., the brain also receives signals from the heart). Amygdala, dmPFC, and insula (among other regions) appear to continually integrate visceral signals into cognition (Allen et al., 2016,^2022^;Critchley et al., 2004;Garfinkel & Critchley, 2016). This interoceptive integration can influence perceptual processes related to anxiety. For instance, the perceived intensity of threat-relevant stimuli may be modulated as a function of the cardiac cycle (Garfinkel et al., 2014). Moreover, when in a state of induced anxiety, there is an alteration in the conscious awareness of internal heart beats (Parrotta et al., 2023). In sum, key threat circuitry (especially amygdala, dmPFC, and insula) in the brain appears to be recruited for both: (1) driving anxiety-dependent alterations in autonomic activity; and (2) monitoring bodily signals.

Although a core framework for studying how anxiety impacts brain-body communication has been developed, one potential issue arises: most of our understanding is driven by tightly controlled experimental paradigms within the confines of a small set of design parameters. The extent to which observed effects are thus seen outside of such conditions remains relatively unknown. As such, we have since extended the research focus to movie fMRI, which consists of presenting movies to subjects while undergoing scanning. By using such unconstrained stimuli, we can test the extent to which prior findings remain consistent across different contexts and translate to more ecologically valid settings (for a critical discussion of the ecological validity of movie-watching paradigms, seeGrall & Finn, 2022).

We have previously validated that suspenseful movies evoke increases in self-reported state anxiety; moreover, the degree to which*state*anxiety is induced appears to vary as a function of individual differences in*trait*anxiety (Kirk et al., 2023). This demonstrates that movie-watching may offer an effective platform for studying anxiety-relevant phenotypes, at least as in terms of self-reported affect in healthy individuals. In regard to studying brain function, it is important that we first build a fundamental understanding of normative responses to movie-evoked anxiety in healthy individuals. By doing so, we can then proceed to study how individuals with pathological anxiety may diverge from such responses. For instance, does brain activity look different in response to suspense compared to traditional anxiety-induction paradigms (e.g., threat of shock)? If so, which regions show discrepancies with prior task-based literature? Little research has sought to address these questions.

Prior work has demonstrated some consistency with threat of shock paradigms: inducing*states*of anxiety through horror movies in healthy volunteers appears to increase amygdala-prefrontal responding (Hudson et al., 2020;Kinreich et al., 2011). In our experiments, we have since demonstrated that suspenseful movies evoke idiosyncratic neural responses pertaining to subclinical trait anxiety (Kirk, Holmes, et al., 2022a,2022b;Kirk, Robinson, et al., 2022). Hence, suspenseful movies appear to offer a novel, effective platform for investigating the neurobiological substrates of anxiety (at least, adaptive, subclinical manifestations of anxiety). Although these studies demonstrate a relationship between anxiety and brain activity, it remains unclear as to what processes might be underpinning these effects. One potential mechanism might be related to communication between the brain and peripheral physiology.

There exist few studies linking neural and autonomic systems during movie-watching. One study using intracranial electrophysiological recordings in humans has provided evidence for a role of insula-dmPFC/anterior cingulate dynamics in shaping cardiac responses to movies (Sonkusare et al., 2023). Moreover, these results indicated these brain-heart responses might possibly be influenced by perceptual features of movies such as emotional dynamics, salience, luminance, and sound. Another study using movie fMRI suggested general arousal/valence-driven heart rate dynamics to be associated with shifts in macro-scale architecture, namely in salience, executive, and default mode networks (which includes, but is not limited to, insula and dmPFC;Young et al., 2017). Yet, to our knowledge, no studies so far have directly investigated whether communication between threat circuitry and the autonomic system alters as a function of anxiety during movie-watching. Therefore, in the present study, we sought to investigate which components of the “defensive response network” may be associated with heart rate during suspenseful movie-watching. We note here that we do not aim to make causal inferences regarding directionality of effects (put simply, whether the brain is influencing the heart and/or the heart is influencing the brain) and our analyses are correlational in nature. As such, in referring to communication between the brain and cardiac activity, we opt for a more theoretically-neutral term, “coherence,” in this paper.

### 1.1 Hypotheses

In the present study, we contrasted brain activation during an anxiogenic, suspenseful clip and a non-suspenseful clip. As stated in our preregistration ( https://osf.io/9vy87/ ), we predicted that the suspenseful movie would be associated with stronger coherence between heart rate and activity in the:

1. Left amygdala.
2. Right amygdala.
3. Left anterior insula.
4. Right anterior insula.
5. dmPFC.

Following hypothesis-testing, we also preregistered to expand our analyses to encapsulate activity and connectivity across a “defensive response network.” In addition to our primary regions of interest for hypothesis-testing, this included hypothalamus, periaqueductal gray, bed nucleus of the stria terminalis, sgACC, and anterior vmPFC.

## 2 Method

### 2.1 Dataset

We made use of the Caltech Conte Center dataset (Kliemann et al., 2022). In brief, n = 29 participants (mean age = 28.8, age SD = 5.3, sex at birth = 18 male 11 female; following exclusion of subjects from the full n = 55, see*Cardiac Data*) watched and listened to a grayscale, 8 min clip from Alfred Hitchock’s “*Bang! You’re Dead*” (Hitchcock, 1961). The clip consists of a child playing with a real, loaded gun, which they (the child) believe is a toy gun. The clip generally builds in suspense until the child almost shoots a maid. We have previously validated that this suspenseful movie clip increases self-reported ratings of state anxiety in an independent sample of 133 healthy volunteers (Cohen’s d = 1.78;Kirk et al., 2023). Additionally, continuously reported state ratings throughout the movie clip correlate highly with independently-rated time series of suspense (*r *= .62), demonstrating a robust link between suspenseful dynamics within the movie clip and induced anxiety. Participants also watched and listened to Pixar’s “*Partly Cloudy*” (Sohn, 2009), an animated short comedy, which served as the non-suspenseful condition. The 5.5 min animated clip consists of anthropomorphized clouds which create baby humans and animals. Participants also underwent 7 min of eyes open resting-state scanning which we used post-hoc as a control condition to contextualize our findings.

Whole-brain scanning was conducted on a Siemens 3T Magnetom Prisma using echo planar imaging: 2.5 mm^2^voxels, TR = 700 ms, TE = 30 ms, FA = 53^o^, and multiband acceleration = 6. Data had already undergone standardized preprocessing (using*fMRIPrep*), including distortion correction, realignment, and coregistration (for full details, seeKliemann et al., 2022).

### 2.2 Analysis

#### 2.2.1 Within-subject processing

##### 2.2.1.1 Cardiac data

**Fig. 1.**
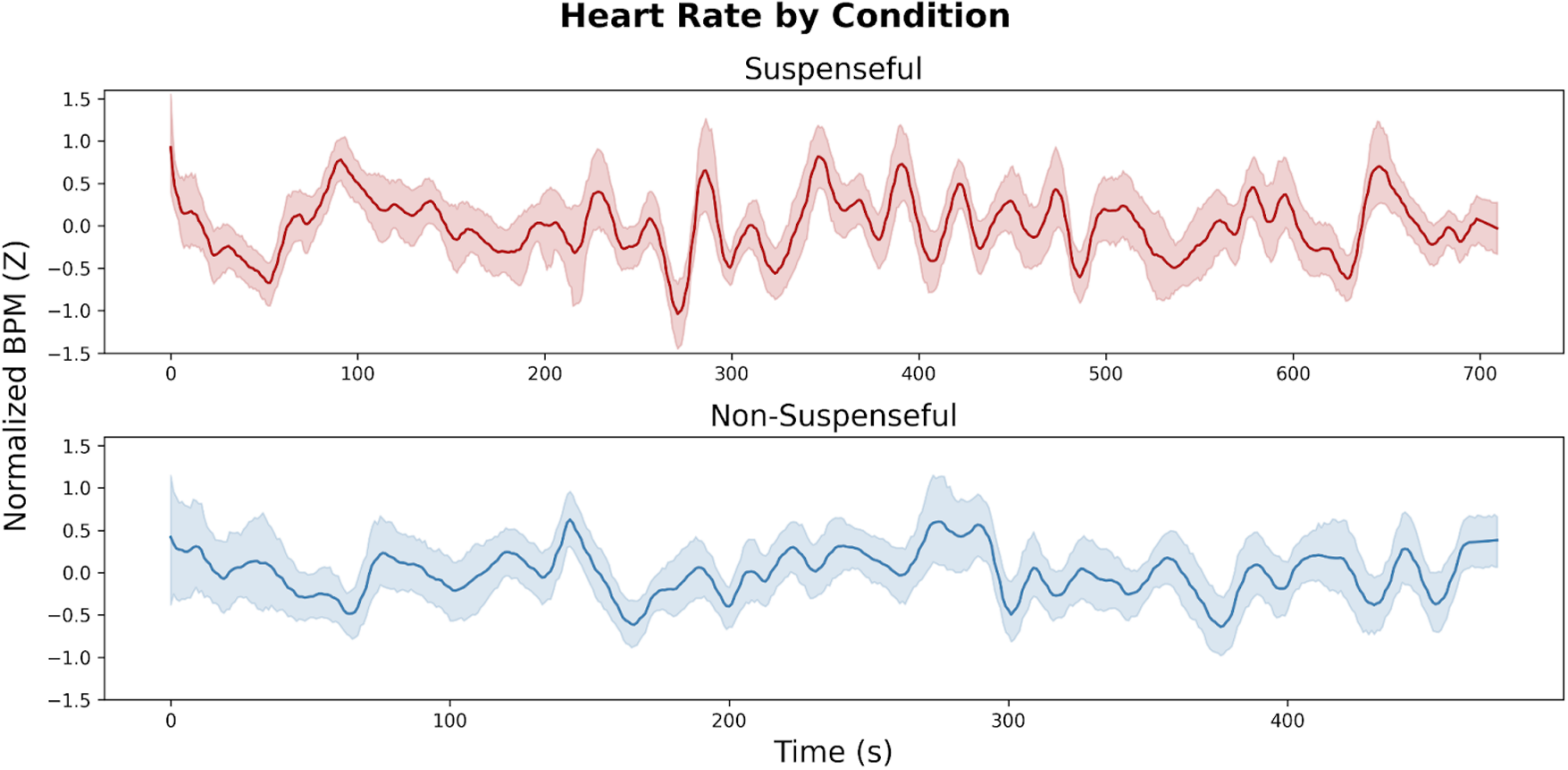
BPM time series averaged across all participants for suspenseful movie-watching (top) and non-suspenseful movie-watching (bottom).

Pulse oximeter data (200 Hz) were previously processed with RapidHRV (Kirk, Bryan, et al., 2022), making use of a 10 s moving window with a 1 s offset. Beats per minute (BPM) constituted our metric for heart rate (figure 1). In this study, we aimed to investigate ongoing, co-fluctuations between the brain and heart (rather than block-wide averages). Our brain-heart coherence measures (see below,*Brain-Heart Coherence*) were based on high-frequency dynamics in brain and cardiac activity in the order of seconds (correlating second-to-second dynamics in brain vs. cardiac timeseries, which had low-frequency trends removed). Therefore, heart rate variability was not used due to its instability at such ultra-short timeframes (Pecchia et al., 2018). Missing data points were interpolated using cubic-spline interpolation (3^rd^order polynomial) and backward/forward fill using the first/last valid data point (for missing data at the beginning/end of the time series). BPM time series were: smoothed using a savitzky-golay filter; upsampled to 700 ms (to match the temporal resolution of fMRI acquisition); detrended (with the same detrending parameters applied to fMRI data, see*fMRI Data*); shifted by 6 s (to account for hemodynamic lag, as used in our previous work;Kirk, Holmes, et al., 2022a); and Z-normalized. RapidHRV output and a priori visual inspection indicated 26 subjects had severe noise present in the signal. These subjects were excluded from the present analysis, leaving 29 subjects for subsequent analyses.

##### 2.2.1.2 fMRI data

We denoised data in AFNI (Cox, 1996; functions and arguments denoted in parentheses) by taking voxel-wise residual time series from a GLM (3dDeconvolve) which contained the following parameters: detrending (-polort “A”; removal of low-frequency drifts with a series of polynomials); nuisance regression of CSF and WM mean signals; and 24 motion parameters (6 rotational and translation + 6 temporal derivatives + 12 squares of raw and derivatives; motion by condition data reported inSupplement 1). For voxel-wise analyses only, we applied smoothing to 6 mm FWHM (3dBlurToFWHM).

**Fig. 2.**
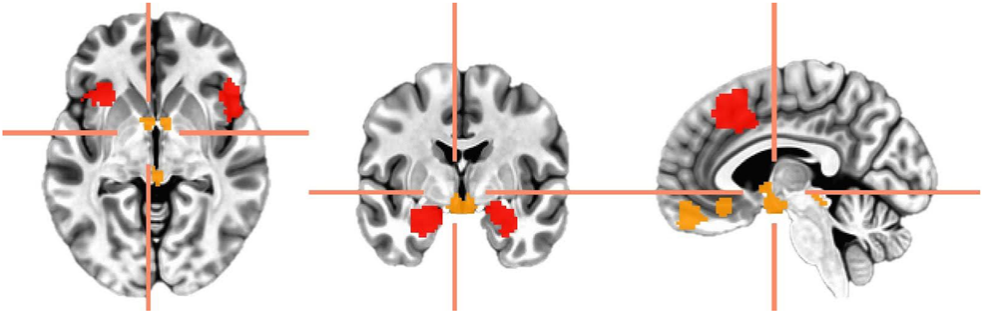
Regions of interest for (MNI x = -4, y = -3, z = -3) primary hypothesis-testing (red) and exploratory analyses (orange). ROIs included are left slice: anterior insula (meta-analytically defined), bed nucleus of the stria terminalis (MNI anatomical mask), and periaqueductal gray (MNI anatomical mask); middle slice: amygdala and hypothalamus (FreeSurfer-defined; i.e., a union of all subjects); right slice: dorsomedial prefrontal cortex, anterior ventromedial prefrontal cortex, and subgenual anterior cortex (meta-analytically defined).

For group-level analyses, time series were extracted (3dmaskave) from 10 regions. For our 3 hypothesis-testing ROIs: dmPFC and anterior insula were defined using meta-analytical clusters (“induced (+) vs. transdiagnostic (+) 20 mm” and “induced (+) vs. pathological anxiety (+) 20 mm” respectively;Chavanne & Robinson, 2021); and amygdala was defined using FreeSurfer parcellations (constrained within an inflated AAL3-defined amygdala MNI mask,Rolls et al., 2020). These specific masks were selected based on our hypotheses, to allow consistency with and comparisons to our prior studies, and because they show spatial convergence with meta-analyses indicative of neural-autonomic communication (Dugré & Potvin, 2023;Ferraro et al., 2022). The remaining 7 ROIs correspond to other regions in the “defensive response network” (hypothalamus, bed nucleus of the stria terminalis, periaqueductal gray, anterior ventromedial prefrontal cortex, and subgenual anterior cingulate cortex;Fig. 2), following the same definition procedures as our previous work (FreeSurfer segmentations for subcortex and meta-analytic clusters for cortex;Billot et al., 2020;Kirk, Holmes, et al., 2022b). For a robustness check, we additionally included the third ventricle defined using the FreeSurfer group average. ROIs were spatially resampled (3dresample) to match the grid spacing of the EPI data.

In addition to activation time series, we derived dynamic connectivity measures across all edges of the “defensive response network.” Using a sliding window-based analysis in the “timecorr” package (width = 29 TRs/20.3 s, gaussian kernel weighting (Owen et al., 2021)), we produced time series of Fisher-transformed correlation coefficients. We selected our window to match that of our prior study (Kirk, Holmes, et al., 2022a; note: supplementary analyses in this prior work suggested changing the window size did not change inference). Time series were then Z-normalized.

##### 2.2.1.3 Brain-heart coherence

Traditionally, ongoing associations between HRF-convolved cardiac measures and the brain signals have been tested by looking at the instantaneous correspondence between the two signals (Nguyen et al., 2016;Valenza et al., 2019). We also took this approach by calculating Fisher transformed bivariate correlations between heart rate and neural (i.e., activity within- and dynamic connectivity across ROIs) time series. However, we also wanted to approach the data in a way which made fewer assumptions concerning the temporal relationship between the two signals. As such, we also made use of dynamic time warping (using “dtaidistance,”Meert et al., 2020), which warped heart rate and neural (activity and dynamic connectivity) time series non-linearly so as to best match all timepoints in regard to similarity, with the final output being a distance measure. Warping paths were constrained using a Sakoe-Chiba band corresponding to 14 TRs (~10 s). We used this constraint so as to focus primarily on relatively quicker-acting brain-heart co-fluctuations which may be related to acute anxiety compared to longer delays which may be underpinned by other biological processes (e.g., digestion and circadian rhythms). Due to the difference in duration between the two conditions, we matched the number of TRs between conditions (i.e., omitting the first section of the suspense condition).

**Fig. 3.**
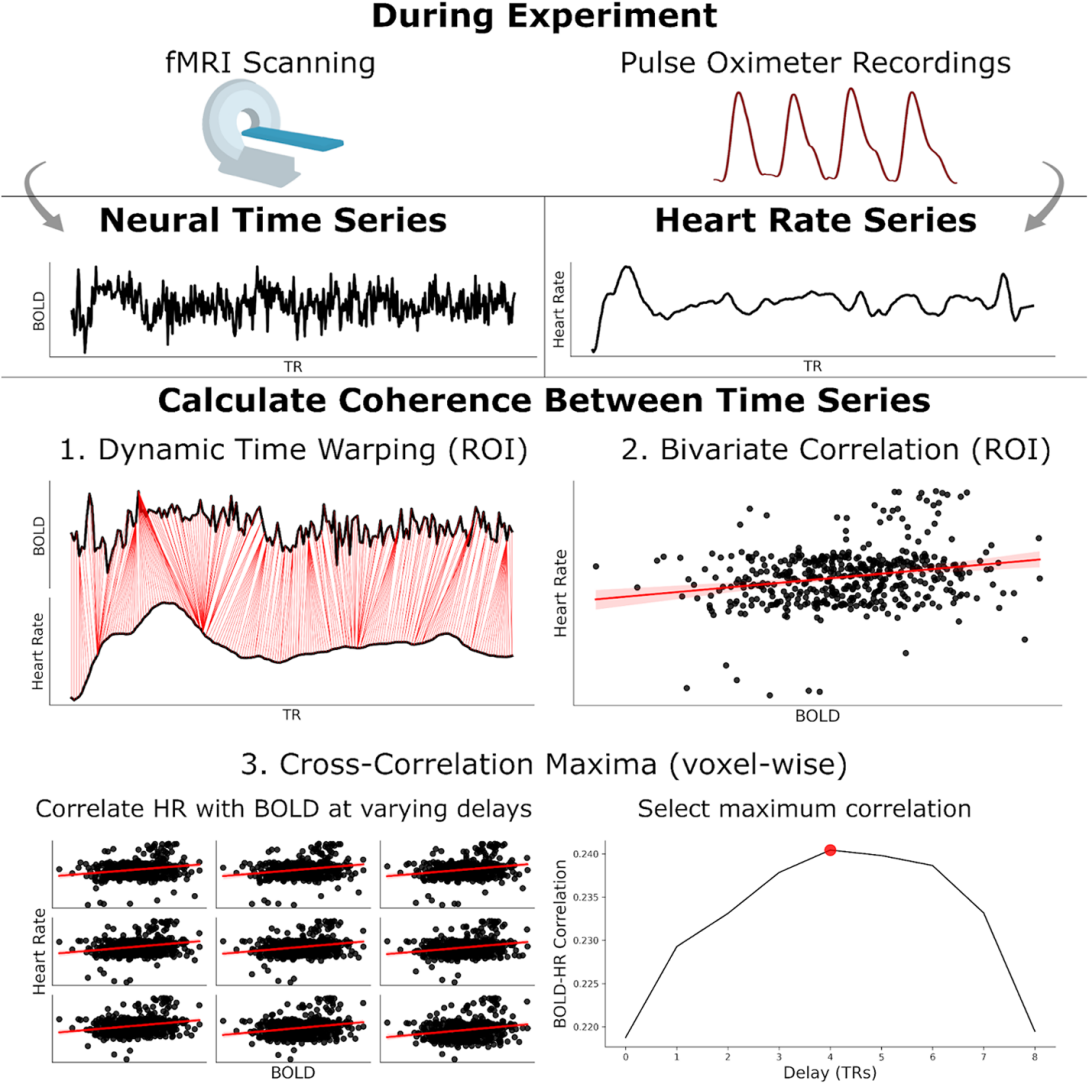
Overview of analysis pipelines. From the fMRI and pulse oximeter signals, we preprocessed and extracted BOLD and heart rate time series. We then calculated the similarity (“coherence”) between these signals using three approaches. For our regions-of-interest analyses, we used (1) dynamic time warping and (2) bivariate correlations. For whole-brain exploratory analyses, we selected (3) cross-correlation maxima at every voxel.

We lastly generated exploratory whole-brain measures. For this, we calculated voxel-wise, Fisher transformed cross-correlation maxima between HR and the BOLD signal (3ddelay;Saad et al., 2001,^2003^;Fig. 3). Here, 3ddelay only tests for lags in BOLD data against the HR signal. As such, we introduced an additional 10 s lag into the HR to allow for tests of neural activity both preceding and following HR. While our ROI-based analyses assumed a canonical HRF delay between BOLD and changes in heart rate (~6 s), this whole-brain analysis was sensitive to BOLD activity preceding changes in heart rate (up to 16 s)*or*changes in heart rate preceding BOLD activity.

#### 2.2.2 Group-level modeling

All group-level models were run in Python and AFNI (Cox, 1996) and used two-tailed tests at*p*< .05. Cardiac data and ROI brain-heart coherence measures were submitted to paired-sample t-tests comparing across suspenseful and non-suspenseful movie conditions as well as during resting-state scanning. Bonferroni correction was run at the level of hypothesis-testing measures (5 activation ROIs,*p*< .01) and exploratory tests (10 activation ROIs + 45 connectivity measures,*p*< .0009). We also conducted contrasts against resting-state measures to help contextualize results.

For our whole-brain analyses, we submitted cross-correlation maxima maps to paired t-tests between conditions (3dttest++). We adjusted for a false positive rate using permutation-based cluster thresholding (-Clustsim, voxel-wise*p*< .001), resulting in a cluster-corrected threshold of k > 16. Coordinates are reported in MNI space.

### 2.3 Deviations from preregistration

We note the following deviations from our preregistration:

- We did not preregister a plan for smoothing fMRI data. This was done post-hoc, but only for exploratory voxel-wise analyses.
- To help contextualize our effects, we have provided additional post-hoc statistics. These include:
  ○ Contrasts between coherence measures derived from movie sessions against resting-state sessions.
  ○ Standalone analyses of cardiac data.
  ○ Descriptive time delay statistics for cross-correlation clusters.
  ○ Additional analyses in ourSupplementary Materials(motion, robustness checks, sex-based differences, correlations with suspense).
- We originally stated 27 subjects were excluded due to excessive noise in the cardiac signal. This was an error, and the actual number of subjects excluded was 26 (leaving 29 for analyses).
- In our voxel-wise analyses, we did not account for the fact that 3ddelay only delays BOLD signals and, in this instance, only tests for HR preceding BOLD activity. As such, we introduced a 10 s lag to account for this.

## 3 Results

### 3.1 Cardiac data

To provide a basic characterization of cardiac data for each condition, we compared unfiltered heart rate between both movies and resting state. Suspenseful movie-watching was associated with significantly higher heart rate (*M *= 67.41,*SD *= 9.50) compared to non-suspenseful movie-watching (*M *= 64.59,*SD *= 9.50;*t*(28) = 2.63,*p *= .014, Cohen’s d = .28), but not compared to resting-state (*M *= 66.53,*SD *= 9.30;*t*(28) = .82,*p *= .42). We did not detect a significant difference between heart rate during the non-suspenseful movie clip versus resting state (*t*(28) = -1.74,*p *= .09). We did not find evidence of differences in heart rate variability (Supplement 2).

### 3.2 Brain-heart coherence

#### 3.2.1 ROI analyses

We first ran paired-sample t-tests on brain-heart coherence measures between the suspenseful and non-suspenseful conditions in our primary regions of interest. For our key hypotheses, we failed to find evidence for a significant difference for either our primary (dynamic time warping) or secondary (bivariate correlation) measures of brain-heart coherence (*p*> .1).

**Fig. 4.**
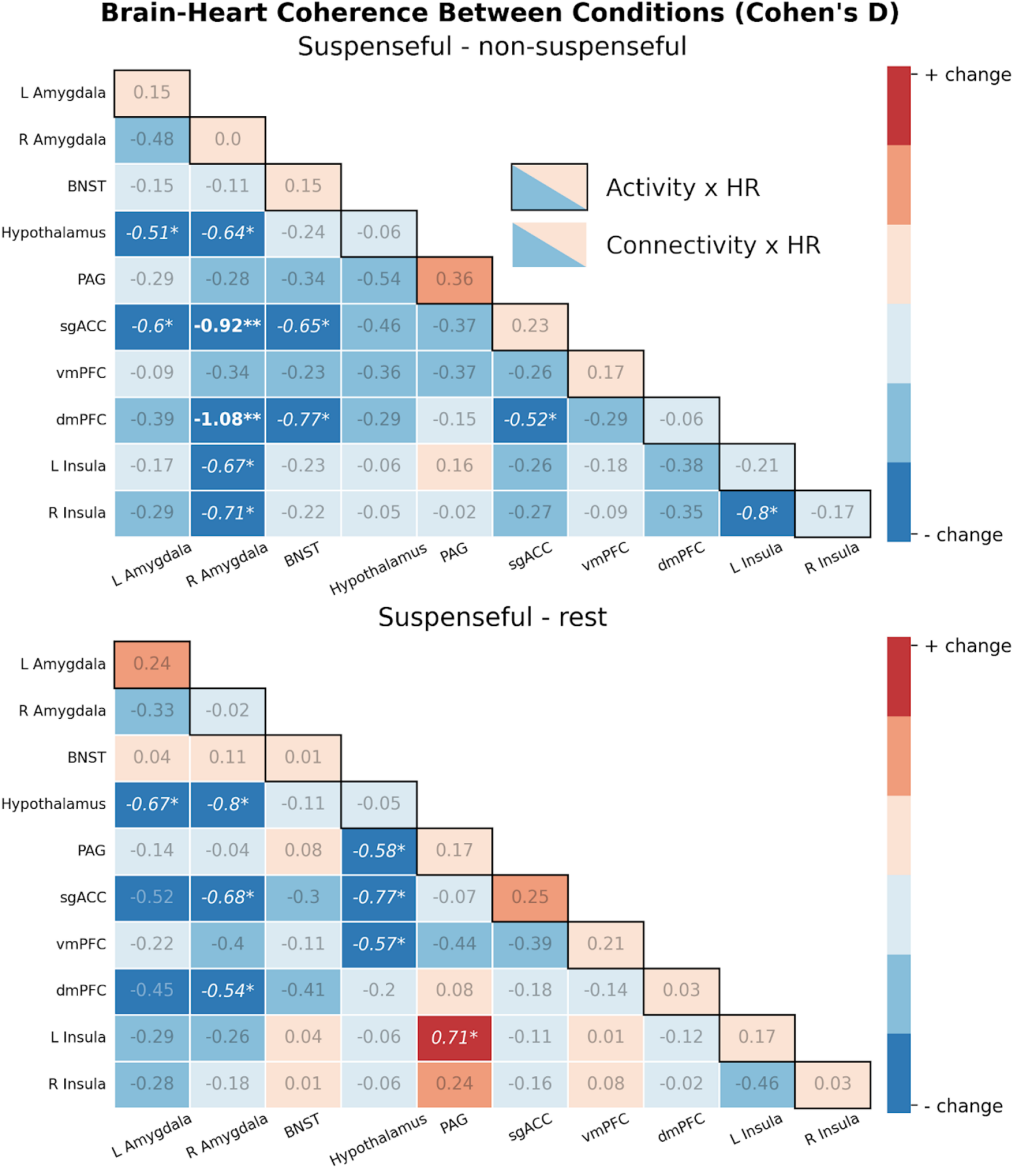
Effect (Cohen’s d) of conditions on brain-heart coherence. Coherence measures in each condition were defined as the Fisher-transformed bivariate correlation between 6 s-lagged heart rate against brain activity/dynamic connectivity. The diagonal of the matrix represents change in coherence between heart rate and activity; below the diagonal represents change in coherence between heart rate and dynamic connectivity. Blue cells indicate suspenseful movie-watching is associated with reduced brain-heart coherence, while red cells indicate increased. Here, we found that the coherence between heart rate and amygdala-prefrontal responding (i.e., right amygdala-dmPFC dynamic connectivity and right amygdala-sgACC dynamic connectivity) was reduced during the suspenseful movie compared to non-suspenseful movie and rest. **p< .0009 (Bonferroni-corrected threshold), *p< .05 (uncorrected).

**Fig. 5.**
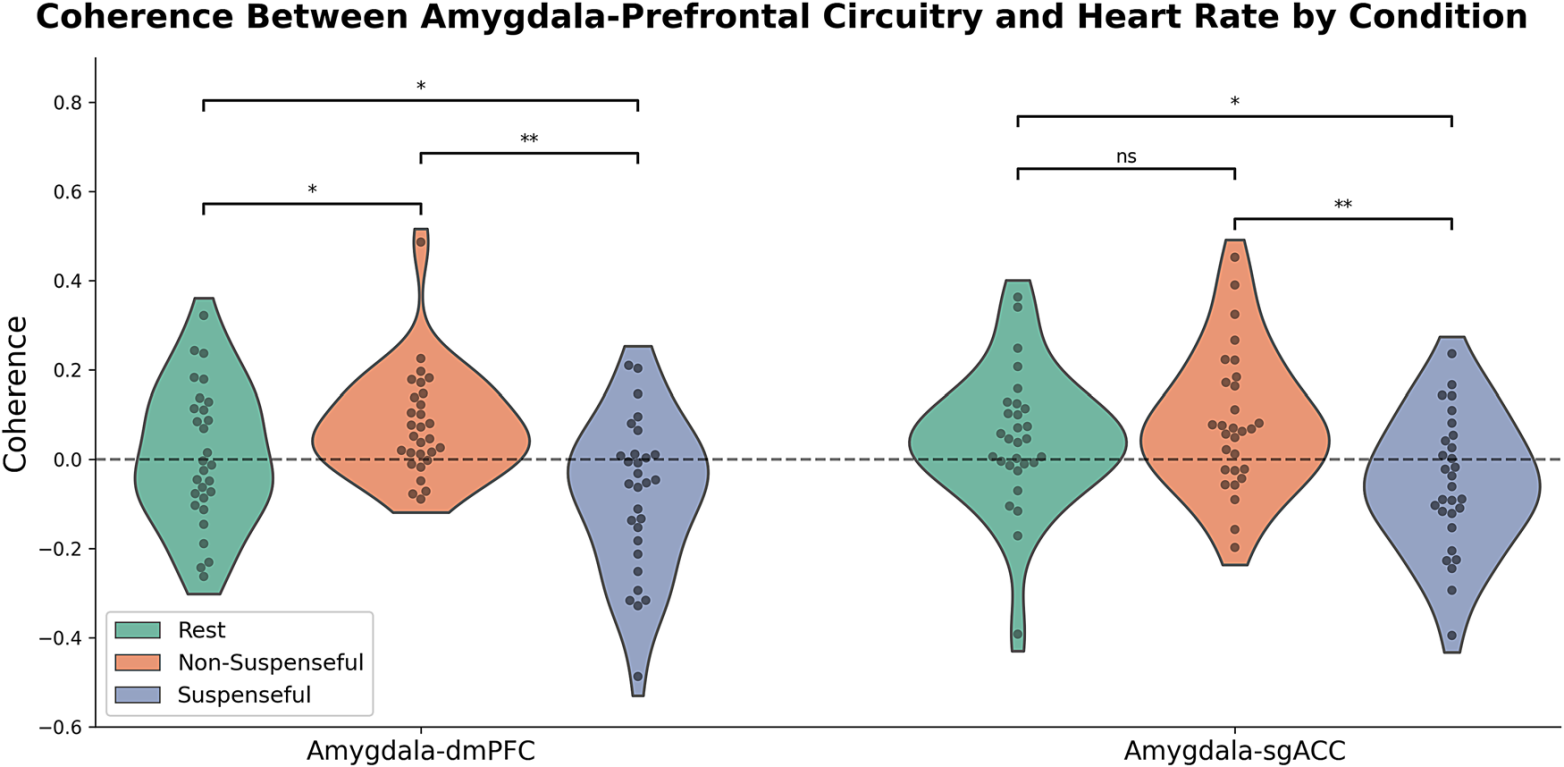
Violin plots detailing coherence (Fisher-transformed correlation coefficients) between amygdala-prefrontal dynamic connectivity and heart rate as participants watched suspenseful/non-suspenseful movie clips or were at rest. This illustrates that coherence was significantly positive during the non-suspenseful condition and was reduced during the suspenseful condition (to negative coherence for amygdala-dmPFC and to non-significant coherence for amygdala-sgACC). *p< .05, **p< .0009.

In our planned exploratory analyses, we expanded this out to a wider “defensive response network” which—in addition to more regions of interest—included dynamic connectivity between all regions. We failed to find evidence using our dynamic time warping measure (*p*> .1), but found two Bonferroni-corrected results for our bivariate correlation measure (instantaneous association between BOLD and 6 s-lagged HR). Specifically, within-subject changes in coherence were observed for right amygdala-dmPFC dynamic connectivity (*t*(28) = -4.41,*p*= .0001) and right amygdala-sgACC dynamic connectivity (*t*(28) = -3.79,*p*= .0007;Fig. 4; for the non-suspenseful versus rest contrast, seeSupplement 3). In both cases, this was characterized by a general positive coherence between heart rate and connectivity in the non-suspenseful condition, which was then reduced during the suspense condition (Fig. 5). This overall reduction in coherence was also observed when comparing the suspenseful movie against a separate resting-state scan rather than the non-suspenseful clip (right amygdala-dmPFC:*t*(28) = -2.10,*p *= .045; amygdala-sgACC:*t*(28) = -2.34,*p *= .026). Increased coherence in the non-suspenseful condition was observed when compared to the resting-state session in right amygdala-dmPFC (*t*(28) = 2.36,*p *= .025), but not right amygdala-sgACC (*t*(28) = .99,*p *= .332). Robustness checks did not suggest these effects were driven by physiological noise (i.e., CSF) nor outliers (Supplement 4).

#### 3.2.2 Whole-brain analysis

**Fig. 6.**
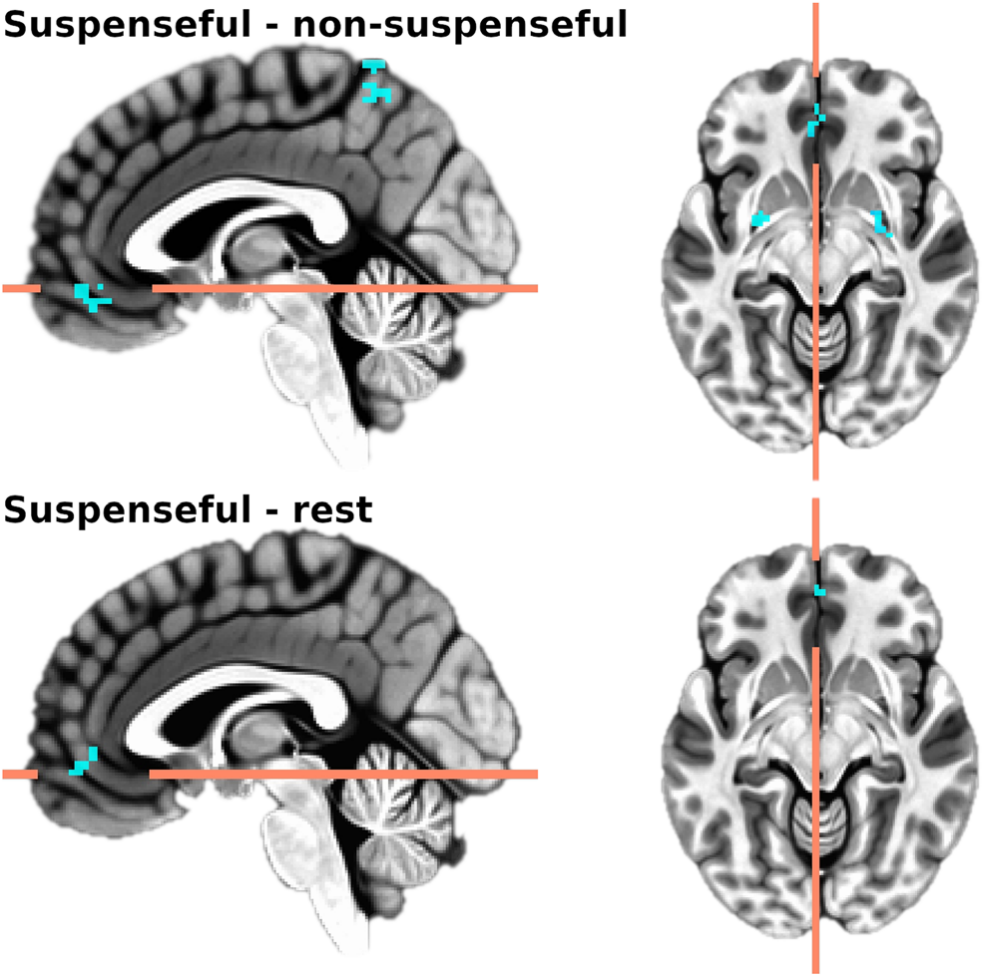
Whole-brain differences in cross-correlation maxima (reduced coherence in suspenseful condition relative to non-suspenseful and rest conditions) projected onto a template brain (MNI x = -2, y = 45, z = -9, voxel-wise*p*< .001, cluster-corrected k > 16). Taking a different approach to our ROI analysis, this technique selected the maximum positive correlation between activity and heart rate across varying lags within each voxel. When contrasting conditions, this suggested coherence between heart rate and activity in precuneus, vmPFC, and bilateral putamen was lower during the suspense condition compared to non-suspense.

Next, we submitted cross-correlation maxima maps to paired-sample t-tests (3dttest++). A different technique to our ROI analyses, this data-driven method finds the maximum, positive correlation between heart rate and BOLD across varying delays (which was applied equally between conditions). Using this approach, we found the positive association between heart rate and activity in 5 regions was lower during the suspenseful movie (vs. non-suspenseful). Of particular relevance, we observed associations in precuneus, vmPFC, and bilateral putamen (Fig. 6; results listed inTable 1). For descriptive purposes, we also extracted mean delay across subjects and conditions in these clusters, which suggested HR was preceding activation by ~16 s. In our post-hoc control analyses, we again found reduced associations between heart rate and brain activity (vmPFC and calcarine gyrus) in the suspense condition compared to rest.

**Table 1.** Whole-brain clusters from cross-correlation maxima maps (suspenseful—non-suspenseful movie conditions).

Region	Voxels	Peak	*p*	Mean delay from HRF-delayed HR to BOLD (95% CI)
Suspenseful—non-suspenseful				
Precuneus	56	[-12, -50, 69]	<.01	17.2 s (12.9, 21.5)
Right cuneus	33	[23, -100, 1]	<.01	14.3 s (10.0, 18.6)
Left putamen	29	[-27, 0, -9]	<.02	16.1 s (11.5, 20.8)
Right putamen	25	[23, 0, -11]	<.02	16.1 s (11.7, 20.5)
VmPFC	25	[-2, 45, -16]	<.02	16.8 s (12.4, 21.2)
Right cerebellum	17	[26, -85, -21]	<.05	21.8 s (16.1, 27.5)
Suspense—rest				
VmPFC	20	[-12, 48, -6]	<.04	23.8 s (18.3, 29.3)
Calcarine gyrus	20	[14, -55, 12]	<.04	18.3 s (13.8, 22.9)

Delay refers to the introduced lag (seconds) in which HR and BOLD achieve the maximum, positive correlation between the two signals. This has been calculated after accounting for a hemodynamic lag of 6 s and refers to the delay from HR to BOLD (i.e., HR preceding BOLD).

## 4 Discussion

Prior literature implicates a moderating role of anxiety on the communication between the brain and peripheral physiology, namely the autonomic nervous system (Critchley, 2005;Makovac et al., 2016). Whether these findings generalize outside of traditional task designs and to relatively more naturalistic settings has yet to be tested. Consequently, we sought to test the extent to which the association between neural responding (BOLD signal) in threat circuitry and cardiac responding (i.e., heart rate) may change as a function of an anxiety-inducing movie clip. Specifically, we tested whether the coherence between neural and cardiac signals differed as participants watched a suspenseful movie clip (anxiety condition), non-suspenseful movie clip, and during rest.

In our primary hypothesis-testing which investigated coherence between heart rate and activity within singular regions, we failed to detect any effects. In our preregistered exploratory analyses, we found evidence for anxiety-relevant alterations in the association between heart rate and: (1) amygdala-dorsomedial prefrontal cortex dynamic connectivity; and (2) amygdala-sgACC dynamic connectivity. Specifically, we observed positive associations between amygdala-prefrontal dynamic connectivity and heart rate in the non-suspenseful condition; this was reduced to negative associations during the suspenseful condition. Moreover, our whole-brain analyses revealed anxiety-relevant reductions in the coherence between heart rate and activity in precuneus, vmPFC, and bilateral putamen. Our general framework posited that states of anxiety would be associated with*increased*brain-heart coherence. This was motivated by the idea that anxiety promotes both top-down signaling to increase sympathetic activity (resulting in faster heart rate), as well as promoting increased interoceptive awareness/sensibility to cardiac activity. However, incongruent with this framework, our results implied coherence was*reduced*in the suspense condition, compared to the non-suspenseful condition. We offer several interpretations.

Firstly, anxiety-dependent sympathetic activation is often coupled with parasympathetic withdrawal, which may occur more rapidly (Critchley, 2009). Our results might be better understood in terms of such withdrawal. Prior research in human and non-human animals has implicated ventral and dorsal sections of the medial prefrontal cortex in the exertion of parasympathetic control (Kim et al., 2011;Resstel et al., 2004;Ter Horst & Postema, 1997;Wong et al., 2007). Moreover, our coherence measures were restricted to relatively short delays (0 s to ~16 s) which may better capture such rapid parasympathetic withdrawal, as opposed to more sluggish fluctuations in sympathetic activity. Therefore, our observations of anxiety-relevant reductions in coherence could be underpinned by amygdala-prefrontal circuits exerting less control over the parasympathetic branch of the autonomic nervous system and thus “releasing the brake” on heart rate.

It is also important to consider that our coherence measures could be sensitive to bottom-up, interoceptive processes. That is, our measures of brain-heart coherence may be driven by the degree to which individuals are attending to their own cardiac signals during movie-watching. Prior work suggests increased awareness of interoceptive signals is associated with increased amygdala-prefrontal responses (i.e., attention to respiration;Doll et al., 2016) and anxiety has typically been thought of as evoking increased interoceptive awareness/sensibility (Mallorquí-Bagué et al., 2016). In the context of the present study, this would lead to the prediction that suspenseful movie-watching would be associated with increased coherence between amygdala-prefrontal responses and heart rate, which we did not find. However, this hinges on the assumption that all forms of induced anxiety evoke increased sensitivity to interoceptive signals. What if suspenseful movie-watching actually detracts from interoceptive integration? Reduced coherence might emerge.

Traditional modalities for anxiety induction, such as threat of shock, are directed at the participant and are designed to pose a perceived physical danger. When there is a physical danger to an individual, increased interoceptive monitoring of physiological state is likely beneficial. Yet, movies do not pose a direct threat to an individual. Instead, they present potential harm to characters. Such outward attention toward others’ potential danger could detract from sensitivity to interoceptive signals (all the while still evoking states of anxiety, as we have previously verified;Kirk et al., 2023). Put simply, because participants are attending to whether another person may be harmed, less attention is oriented toward their own body, resulting in reduced brain-heart communication. However, without any direct measures of interoception nor an active manipulation, it is outside the scope of the current study to draw conclusions regarding directional causality (brain influencing the heart, heart influencing the brain, or causality induced by an external factor). It may therefore be useful for future studies to consider the implementation of interventions (e.g., pharmacological or psychological) or interoceptive biomarkers (e.g., heartbeat evoked potentials using MEG/EEG) to provide a mechanistic characterization of what underpins our coherence measures and thus elucidate the contribution of interoceptive signals to neural responses during movie-watching.

A final consideration is that effects may be driven by our non-suspenseful condition. The non-suspenseful condition, “*Partly Cloudy*,” contains positively-valenced content. Anxiety is not the only emotion to impact autonomic responding. Positively valenced stimuli can still elicit responses in peripheral physiology. For instance, prior research has noted cardiac acceleration in response to happy faces (albeit of a lower magnitude than negatively-valenced faces), and this may correlate with responses in the amygdala (Critchley et al., 2005). It is plausible that our effects do not pertain to*reduced*brain-heart communication in the suspenseful condition; rather,*increased*communication as a function of positively-valenced content in the non-suspenseful condition. However, we argue this is the least likely interpretation as our post-hoc analyses revealed the suspenseful movie clip to still be associated with reduced brain-heart communication when compared to “rest.”

### 4.1 Limitations and future research

It is important to contextualize our significant findings with our non-significant results, as well as our methodological constraints and choices. Although the tests which revealed significant findings were planned a priori (dynamic connectivity and whole-brain analyses), we preregistered these as exploratory tests. Our primary hypotheses concerned activation-based measures within the amygdala, dmPFC, and anterior insula. We did not detect altered coherence between heart rate and activation in these regions across conditions. Thus, we failed to find evidence in support of our key hypotheses. This may represent a true null and effects primarily emerge at the level of communication between amygdala-prefrontal circuitry rather than activity within singular regions. Given the sample size (n = 29) and the neuroimaging measure, it is difficult to provide evidence in favor of the null and the presented results should be considered preliminary. We therefore recommend future work to test whether anxiety-relevant alterations in neural-autonomic communication do primarily emerge at the level of connectivity as opposed to regional activity (at least, in relation to amygdala-prefrontal circuitry).

Replication of these results is especially important given the exploratory nature of our coherence measures, which used varying methods to approximate brain-heart coherence (dynamic time warping, bivariate correlations, and cross-correlation maxima). Our measures were either predominantly data-driven (dynamic time warping and cross-correlation maxima) or assumed instantaneous correspondence between BOLD and HRF-lagged heart rate (bivariate correlation). It will be useful for future work to better characterize the temporal relationship between ongoing neural activity (BOLD) and cardiac responding in these contexts. For instance, methods such as vector autoregression may help provide a general overview for how BOLD and HR co-fluctuate at varying time scales during movie-watching; moreover, these models may be able to assess whether anxiety appears to impact communication at specific lags. Following this, research into brain-heart communication may be able to produce measures more sensitive to the effects of induced anxiety and holds potential to disentangle associations with sympathetic activity, parasympathetic activity, and interoception.

A key challenge with our study was appropriately assessing brain-heart coherence in the context of the imaging modality. fMRI is inherently underpinned by cardiac responses, including heart rate (Shmueli et al., 2007), and contains sources of noises relating to physiology such as vasculature and respiration (Birn et al., 2008;Chang et al., 2008). We attempted to control for the impact of physiological confounds by denoising physiological signals from the fMRI data, contrasting brain-heart coherence between multiple conditions, calculating measures via co-fluctuations in HR and brain activity*within*conditions (rather than stimuli-averaged signal intensities which would be intrinsically confounded by HR), and through robustness tests. Nonetheless, we cannot be certain that there are no physiological confounds present in our analyses. As such, we advise that validation with other imaging modalities and proxies for autonomic responding (e.g., skin conductance) is needed before inferring a robust relationship.

Generalization of our results to other movie stimuli is also strongly encouraged. Here, we conducted the present study in order to provide generalizability from the anxiety literature to a relatively more naturalistic platform. However, the unconstrained nature of the movie paradigm used here limits the ability to delineate how specific features of the stimuli influence coherence measures. We have previously noted significant collinearity between audiovisual sensory features and suspense in the movie stimuli used here (Kirk, Holmes, et al., 2022a). Such collinearity is often present in movies due to artistic choices designed to elicit affective states (Grall & Finn, 2022); for example, music and lighting may be used to shift moods between pleasant and unpleasant. Relevant for our analyses, one clip was a live-action, black and white clip with sparse intervals of music, while the other was an animated, color clip with music throughout. As features such as music have the potential to influence autonomic balance in the absence of induced anxiety (Mojtabavi et al., 2020), we encourage future work to validate our findings with movie stimuli which vary in collinearity among sensory features. Moreover, the two movie stimuli appear to differ in valence, but were not purposefully matched in arousal (albeit, we could not formally test this with the available data due to the lack of valence/arousal ratings). Whether these changes in brain-heart coherence are specific to induced states of anxiety or general arousal cannot be inferred within the present study. Future work utilizing clips with both positive valence and high arousal as a control will be needed to specify the unique contributions of induced anxiety (vs. general arousal) to brain-heart coherence during movie-watching.

The present study used a dataset which included 29 subjects watching short movie clips (<10 min per condition). It will be important to test whether these effects hold when using larger and longer samples, due to the potential for instability of effects in small sample sizes with short scans (Birn et al., 2013;Marek et al., 2022). We also encourage future research to consider using scanning parameters with smaller voxel sizes. Our analytical pipeline used averaged time series across entire regions. However, it is important to caveat this with the fact that regions vary in function across space. For instance, the amygdala has sub-nuclei with various projections that perform a variety of anxiety-relevant functions (Pessoa, 2010). Similarly, we only covered an anterior section of the insula (which may preclude capturing some interoceptive-relevant activity in posterior insula;(Bud) Craig, 2003;Nguyen et al., 2016). Suspenseful movie fMRI data acquired with finer grained spatial resolutions may enable researchers to look at the contributions of specific subregions (e.g., basolateral vs. central amygdala and anterior vs. posterior insula) to anxiety-relevant alterations in brain-heart communication.

Finally, our present findings need to be contextualized entirely in terms of induced states of adaptive anxiety in a subclinical sample. Future work should seek to investigate how pathological levels of anxiety may impact brain-heart communication during movie-watching. We encourage researchers to test whether suspense-elicited alterations in brain-heart coherence remain intact, exaggerated, or show an inverse relationship in individuals with anxiety disorders. Moreover, given known interactions between autonomic balance, age, and sex (Alvares et al., 2013;Mutz et al., 2022), explore how these effects might interact with individual differences in demographic features (seeSupplement 5for our data split by sex).

## 5 Conclusion

We aimed to investigate how coherence between the brain and the heart may change as a function of an anxiety-inducing, suspenseful movie-watching. Here, we found preliminary evidence for anxiety-relevant alterations in the coherence between heart rate and amygdala-prefrontal dynamic connectivity (amygdala-dmPFC and amygdala-sgACC). However, effects were in the inverse direction to which we hypothesized. Brain-heart coherence was positive during the non-suspenseful condition, but reduced during the suspenseful condition. This effect remained consistent when we contrasted the suspenseful movie condition to rest. We also found evidence for reduced coherence between heart rate and activity in precuneus, vmPFC, and bilateral putamen, but these effects were not always consistent when controlling for coherence at rest. We posit that anxiety-relevant decreases in brain-heart coherence may be underpinned by parasympathetic withdrawal or decreased interoceptive awareness during suspenseful movie-watching.

## Data and Code Availability

The Caltech Conte Center multimodal data resource (Kliemann et al., 2022), which was approved by the institutional review board at the California Institute of Technology, is available through OpenNeuro (https://openneuro.org/datasets/ds003798). Upon publication, all code will be available on our Open Science Framework page for this study (https://osf.io/9vy87/).

## Author Contributions

P.A.K.: Conceptualization, methodology, formal analysis, visualization, writing—original draft, and writing—review & editing. O.J.R.: Methodology, writing—review & editing, and supervision.

## Funding

P.A.K. was supported by the Leverhulme Trust (Grant/Award Number: DS-2017-026). O.J.R. is supported by the Medical Research Council (Grant/Award Number: MR/R020817/1).

## Declaration of Competing Interest

O.J.R.’s MRC senior fellowship is partially in collaboration with Cambridge Cognition and he ran an investigator-initiated trial with medication donated by Lundbeck. He also held an MRC Proximity to discovery award with Roche regarding work on heart rate variability and anxiety. He has also completed consultancy work for Peak, Blackthorn therapeutics, Roche, and IESO digital health. P.A.K. was previously employed by Roche for biomarker research on remote sensing and stress.

## Supplementary Material

Supplementary Material
