# Supplementary material for: Preliminary evidence for altered brain-heart coherence during anxiogenic movies
Source: Imaging Neurosci (Camb). 2024 May 2;2:imag-2-00156. doi: 10.1162/imag_a_00156 (PMC12247548; doi:10.1162/imag_a_00156)
Supplement: Supplementary Material [file imag_a_00156-supp.pdf]

Supplementary Materials

Preliminary evidence for altered brain-heart coherence during anxiogenic movies

Peter A. Kirk & Oliver J. Robinson

Supplement 1. Motion by condition.

We did not detect a significant difference in motion (average framewise displacement) between: suspenseful (M=0.14, SD=0.05) and non-suspenseful conditions (M=0.14, SD=0.05; $t(28)=0.80$, $p=.444$); suspenseful vs resting-state (M=0.14, SD=0.05, $t(28)=0.26$, $p=.796$); or non-suspenseful vs resting-state ($t(28)=-0.49$, $p=.628$).

Supplement 2. Additional analyses of cardiac data.

In addition to heart rate measures, we conducted post-hoc analyses of heart rate variability data using both time-domain (RMSSD) and frequency-domain (HF power) measures. We did not detect significant differences between: suspenseful vs non-suspenseful (RMSSD: $t(28)=-1.62$, $p=.117$; HF: $t(28)=-1.08$, $p=.290$); suspenseful vs resting-state (RMSSD: $t(28)=0.97$, $p=.343$; HF: $t(28)=0.48$, $p=.638$); and non-suspenseful vs resting-state conditions (RMSSD: $t(28)=1.58$, $p=.126$; HF: $t(28)=1.292$, $p=.268$).

Supplement 3. Non-suspenseful vs resting-state contrast.

Brain-heart coherence (as determined by bivariate correlation) between non-suspenseful vs resting-state (figure S1). The diagonal of the matrix represents change in coherence between heart rate and activity; below the diagonal represents change in coherence between heart rate and dynamic connectivity. Blue cells indicate suspenseful movie-watching is associated with reduced brain-heart coherence, while red cells indicate increased. **p* < .05 (uncorrected). None of these effects survive Bonferroni correction. However, we note that uncorrected significant results (e.g., HR x amygdala-dmPFC connectivity) suggest coherence might be higher during the non-suspenseful condition compared to resting-state. Due to the anxiogenic nature of resting-state scanning (Munn & Jordan, 2011, *JBI Libr Syst Rev.*, doi.org/10.11124/01938924-201109190-00001) we suggest these results are congruent with our suspenseful vs non-suspenseful contrast. Put simply, the non-suspenseful clip may provide an anxiolytic effect, and thus we see the inverse effect of our suspenseful vs resting-state comparisons.

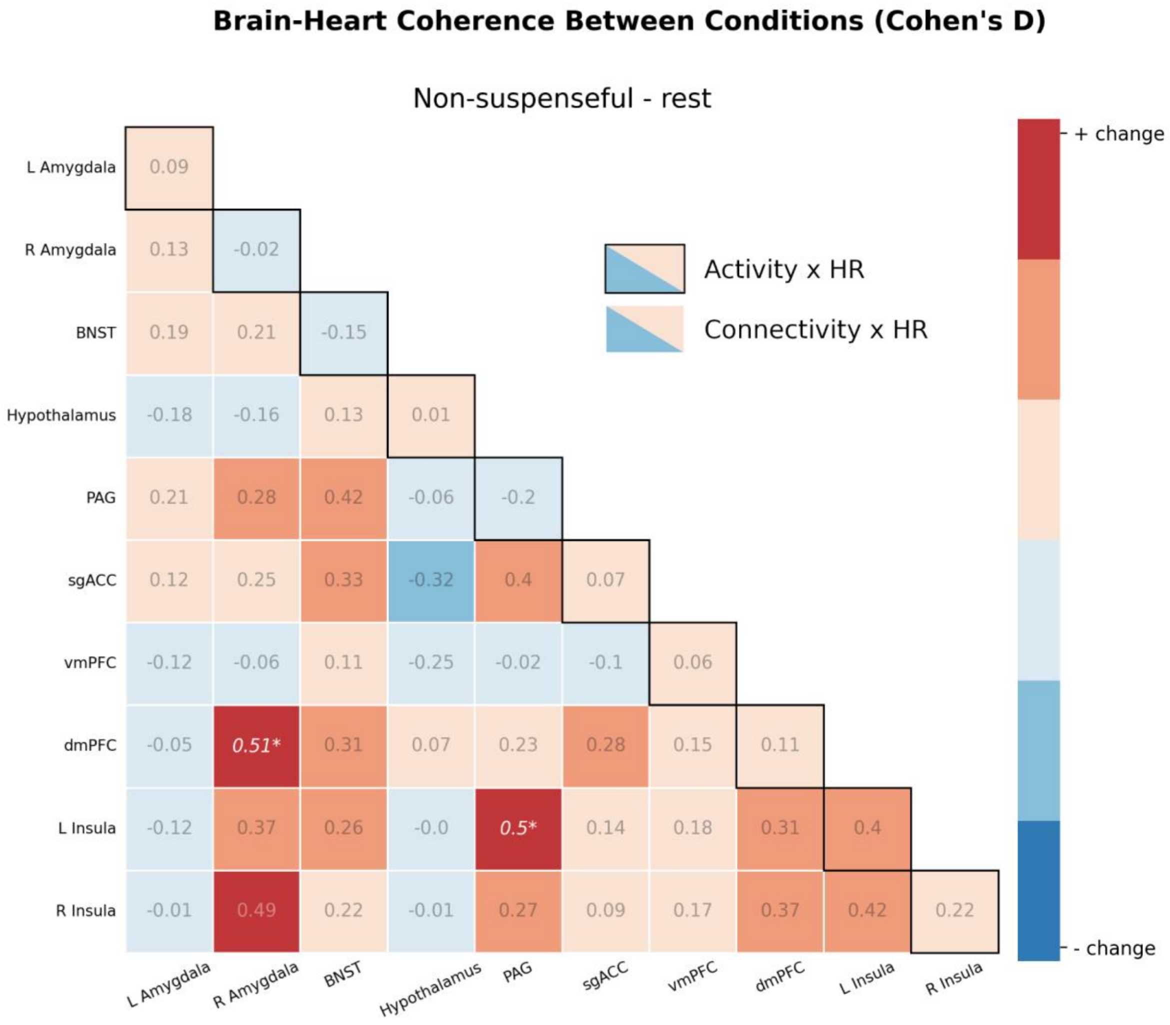

Figure S1. Difference (Cohen's d) in brain-heart coherence between non-suspenseful and rest conditions. **p* < .05 (uncorrected).

Supplement 4. Robustness checks.

As an additional robustness check for our results, we assessed altered 'coherence' between heart rate and right amygdala-third ventricle 'dynamic connectivity' to test whether physiological noise in the BOLD signal may be underpinning these effects (despite having regressed out WM and CSF signals in denoising). The coherence between heart rate and amygdala-ventricle 'dynamic connectivity' was not significantly different between suspenseful and non-suspenseful conditions ($t(28)=-1.28$, $p=.212$), nor suspenseful and resting-state ($t(28)=-.65$, $p=.519$), lending support for the notion that our previous results may be driven by neural activity.

We also tested the extent to which alterations in brain-heart coherence could be driven by outliers. As such, we re-ran analyses following exclusion of participants whose coherence measures exceeded 3 median absolute deviations (2 and 4 participants excluded for amygdala-dmPFC and amygdala-sgACC coherence measures respectively). Specifically, coherence between heart rate and right amygdala-dmPFC connectivity was still significantly lower in the suspenseful condition compared to the non-suspenseful condition ($t(26)=-4.16$, $p=.0003$). Likewise, coherence between heart rate and right amygdala-sgACC connectivity was still significantly lower in the suspenseful condition compared to the non-suspenseful condition ($t(26)=-3.51$, $p=.002$).

Supplement 5. Sex-based differences.

We conducted a series of Welch's t-tests to investigate sex-based differences in cardiac and coherence measures. We did not detect a significant effect of sex at birth on change in heart rate for: suspenseful vs non-suspenseful conditions (mean ΔBPM: male=3.37, female=1.92; $t(26.77)=0.74$, $p=.466$); nor during rest (mean BPM: male = 65.23, female = 28.65; $t(26.24)=-1.09$, $p=.284$). We did not detect a significant effect of sex at birth on change in brain-heart coherence (suspense vs non-suspenseful) derived through: amygdala-dmPFC connectivity ($t(23.59)=-1.34$, $p=.192$) nor amygdala-sgACC connectivity ($t(26.83)=-1.49$, $p=.148$). We did not detect sex-based differences in resting-state derived coherence between HR and: amygdala-dmPFC connectivity ($t(18.46)=0.50$, $p=.625$) or amygdala-sgACC connectivity ($t(21.52)=-0.99$, $p=.334$). Below we provide violin plots detailing coherence (Fisher-transformed correlation coefficients) between amygdala-prefrontal dynamic connectivity and heart rate across conditions split by sex at birth (figure S2).

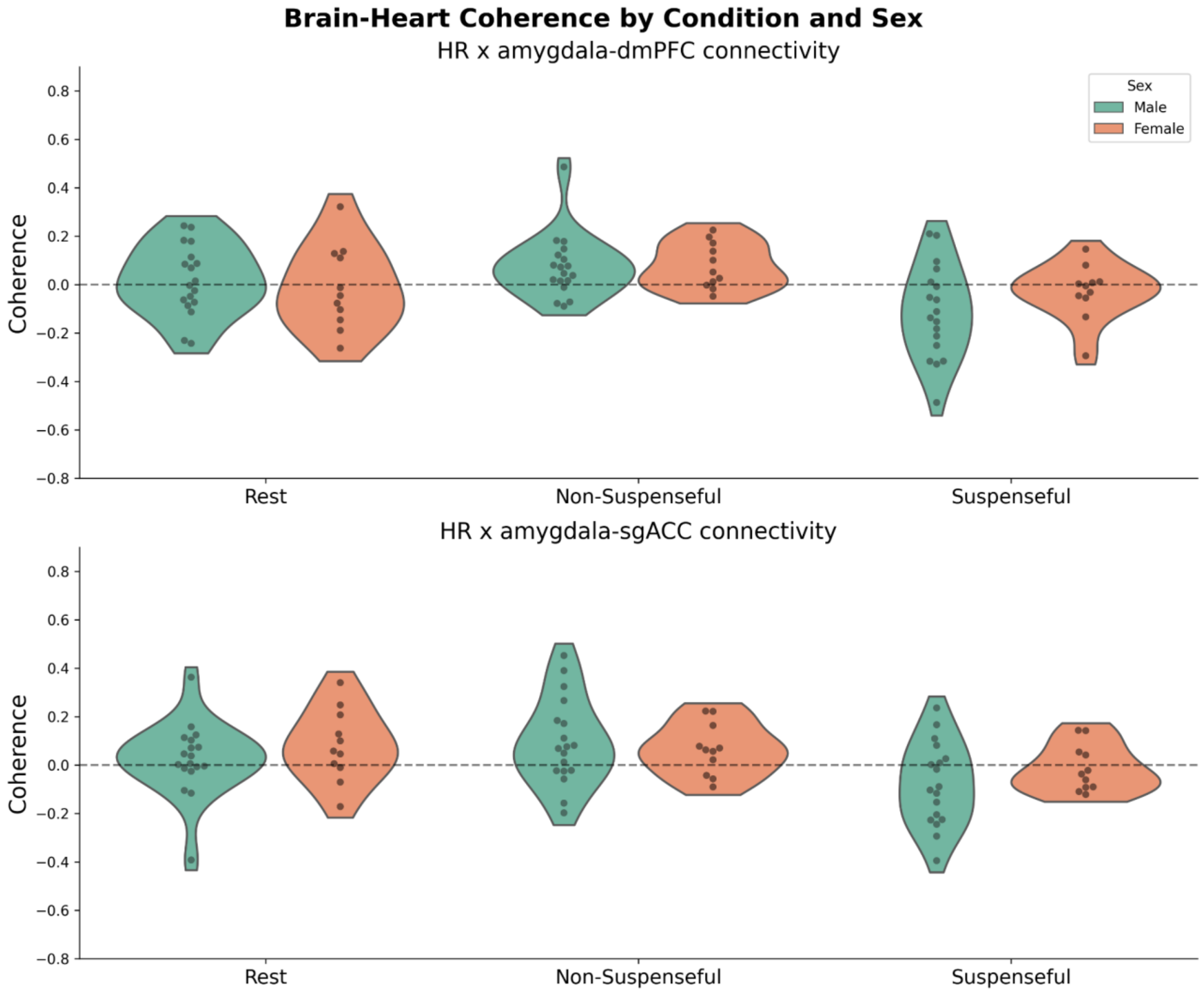

Figure S2. Violin plots detailing coherence (Fisher-transformed correlation coefficients) between amygdala-prefrontal dynamic connectivity and heart rate as participants watched suspenseful/non-suspenseful movie clips or were at rest, split by sex.

Supplement 6. Dynamic brain-heart coherence x Suspense.

In addition to our planned analyses, we ran a post-hoc analysis which looked at the association between ongoing fluctuations in brain-heart coherence and time series of continuous ratings of state anxiety during the suspenseful movie clip we acquired and averaged from 133 participants in a different study (https://osf.io/preprints/psyarxiv/dqhnx). The direction of effects were the same but not significant.

amygdala-dmPFC x HR x state anxiety: $t(28)=-1.30$, $p=0.204$ CI95%=[-0.09, 0.02].

amygdala-sgACC x HR x state anxiety: $t(28)=-0.85$, $p=0.404$ CI95%=[-0.08, 0.03].

This was not included in the main results section as it used: (1) a sliding-window, dynamic correlation of two BOLD signals, followed by (2) a sliding-window, dynamic correlation with heart rate; followed by (3) a correlation with independent, subjective ratings of induced anxiety from a different sample. The dynamic brain-heart coherence measures (outcome of step 2) are thus quite noisy (figure S3). This is likely because it was generated using a smaller number of data points (i.e., 20s/29 TRs to match dynamic connectivity) compared to our block-wide measures which makes use of the entire time series.

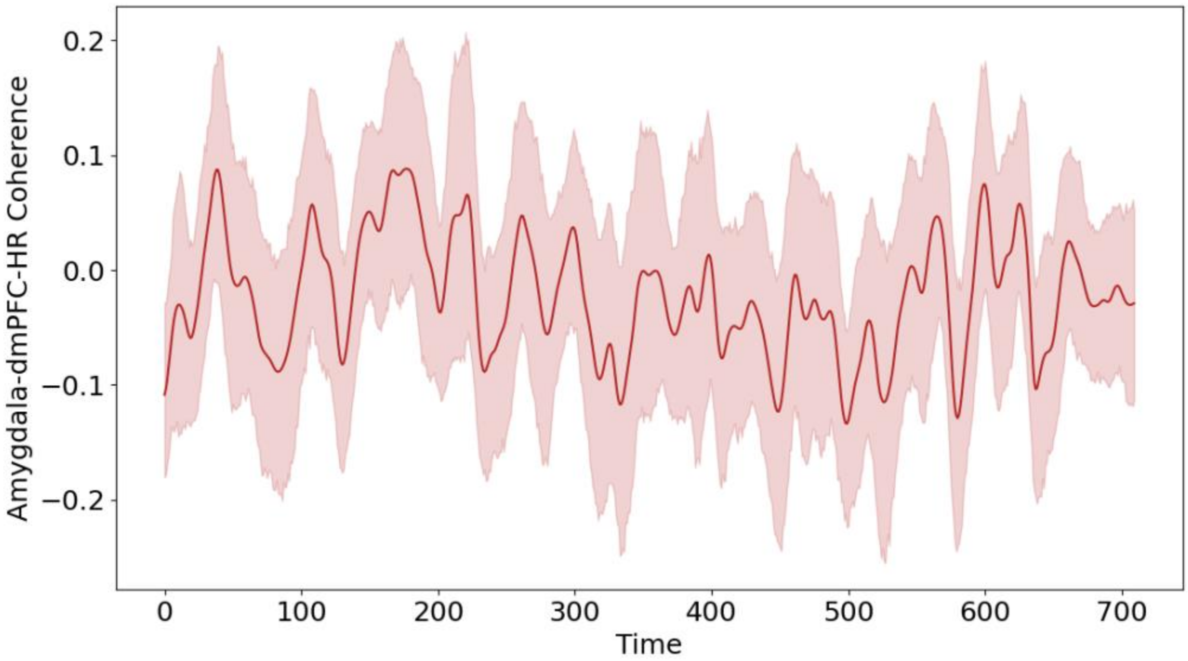

Figure S3. Dynamic coherence between heart rate and amygdala-dmPFC dynamic connectivity.
